# Molar-Based Targeted Metabolic Profiling of Cyanobacterial Strains with Potential for Biological Production

**DOI:** 10.3390/metabo4020499

**Published:** 2014-06-20

**Authors:** Yudai Dempo, Erika Ohta, Yasumune Nakayama, Takeshi Bamba, Eiichiro Fukusaki

**Affiliations:** Department of Biotechnology, Graduate School of Engineering, Osaka University, 2-1 Yamadaoka, Suita, Osaka 565-0871, Japan; E-Mails: yudai_dempo@bio.eng.osaka-u.ac.jp (Y.D.); erika_ohta@bio.eng.osaka-u.ac.jp (E.O.); yasumune_nakayama@bio.eng.osaka-u.ac.jp (Y.N.); bamba@bio.eng.osaka-u.ac.jp (T.B.)

**Keywords:** metabolomics, cyanobacteria, biological production, absolute quantitation, triple quadrupole mass spectrometry

## Abstract

Recently, cyanobacteria have become one of the most attractive hosts for biochemical production due to its high proliferative ability and ease of genetic manipulation. Several researches aimed at biological production using modified cyanobacteria have been reported previously. However, to improve the yield of bioproducts, a thorough understanding of the intercellular metabolism of cyanobacteria is necessary. Metabolic profiling techniques have proven to be powerful tools for monitoring cellular metabolism of various organisms and can be applied to elucidate the details of cyanobacterial metabolism. In this study, we constructed a metabolic profiling method for cyanobacteria using ^13^C-labeled cell extracts as internal standards. Using this method, absolute concentrations of 84 metabolites were successfully determined in three cyanobacterial strains which are commonly used as background strains for metabolic engineering. By comparing the differences in basic metabolic potentials of the three cyanobacterial strains, we found a well-correlated relationship between intracellular energy state and growth in cyanobacteria. By integrating our results with the previously reported biological production pathways in cyanobacteria, we found putative limiting step of carbon flux. The information obtained from this study will not only help gain insights in cyanobacterial physiology but also serve as a foundation for future metabolic engineering studies using cyanobacteria.

## 1. Introduction

Cyanobacteria are ubiquitous, globally important photosynthetic microorganisms that are capable of providing oxygen to the atmosphere by using solar energy. Recently, photosynthetic microorganisms have been considered as attractive candidates for biomass resources due to their high photosynthetic ability. In particular, cyanobacterial biofuel production derived from environmental CO_2_ has been considered as one of the effective ways to reduce CO_2_ emissions and construct a sustainable low-carbon society system. Cyanobacteria naturally produce valuable materials including lipids [1], sugars [2] and some kinds of polymers [3] directly from atmospheric CO_2_ although they do not have the inherent ability to produce industrially important materials such as higher alcohols. However, currently available tools for genetic modification allowed metabolic engineers to use cyanobacterium as a host for inserting exogenous genes for production of industrially important compounds. In fact, recent researches in cyanobacterial biological production have successfully achieved the production of targeted materials such as fatty acids [1], sugars [2], hydrogen [4], acetone [5], mannitol [6], ethylene [7,8], ethanol [9], isoprene [10], 3-hydroxybutyrate [11], 2,3-butanediol [12], isobutyraldehyde, isobutanol [13], isopropanol [14], 1-butanol [15], and 2-methy-1-butanol [16]. However, most of the bioprocess trials were not satisfactory for industrial application. An inadequate systematic metabolome catalogue of commonly used cyanobacterial strains might cause misunderstanding of the metabolome state, which in turn may lead to a misguided metabolic engineering strategy. Therefore, a robust metabolic profiling system of cyanobacteria must be initially established.

Metabolic profiling has been established as a powerful tool for gaining insights in cellular metabolism [17,18]. Although relative metabolite concentration changes can be informative, levels of absolute metabolite concentrations can be critical. Especially for understanding metabolic dynamics, it is a prerequisite for several approaches in metabolic analysis [19]. In particular, this information is useful for strain improvement via metabolic engineering as it is important to monitor actual changes in the altered cellular metabolism after gene manipulation [20,21]. Recently, advances in chromatography and mass spectrometry technologies have allowed metabolomics to play a more important role in biological studies since they enabled simultaneous measurements of numerous cellular metabolites. As a result, these technologies are increasingly being widely applied in mapping the changes in altered or natural cellular metabolisms.

Currently, the cyanobacterial strains *Synechococcus elongatus* PCC7942, *Synechococcus* sp. PCC7002, and *Synechocystis* sp. PCC6803 (hereafter PCC7942, PCC7002, PCC6803, respectively) are most often used as background strains for metabolic engineering since the whole genome sequence and established tools for gene manipulation are available. However, without major phenotypic differences and available metabolome information of candidate strains, the choice of background strain will probably rely subjectively on the researcher's experience and intuition.

In this study, therefore, we introduced an analytical procedure for molar-based widely targeted metabolic profiling analysis to characterize the difference in the metabolomes of three cyanobacterial strains with potential for biological production namely PCC7942, PCC7002, and PCC6803. To perform widely targeted metabolic profiling in the multiple reaction monitoring (MRM) mode, gas chromatography–triple quadrupole mass spectrometry (GC/QqQ-MS) was used for analyzing amino acids and organic acids, while analysis of intermediates of central metabolism such as sugar phosphates and cofactors was performed using reserved phase ion-pair liquid chromatography (RP-IP-LC)/QqQ-MS. Triple quadrupole mass spectrometer is suitable for metabolic profiling because of its high sensitivity and selectivity as well as its wide dynamic range that makes it possible to give accurate quantitative information [22,23]. Moreover, to obtain the absolute concentration of each metabolite, we used ^13^C-labeled cell extracts as internal standards, a technique known to effectively reduce errors due to variations occurring in the analysis and sample processing [24]. This method is restricted to studies using culturable organisms because all metabolites in the internal standard need to be fully or partially labeled. However, when dealing with a large number of samples, our method is expected to be less tedious than other high accuracy quantitative methods such as standard addition method. Finally, we compared and discussed the molar-based differences in the cellular metabolism of the three cyanobacterial strains in their mid-exponential phase. These results provide informative data and an objective viewpoint for further metabolic engineering researches in cyanobacteria [25].

## 2. Results and Discussion

### 2.1. Optimization of ^13^C-internal Standard Amount for Quantitation

Regarding the internal standard, equal or close peak intensities of targeted analytes are desirable. Here, we use the uniformly ^13^C-labeled cell extracts as the internal standard and their preparation method is described in the Experimental Section. To determine the appropriate ^13^C-IS concentration, four different amounts of ^13^C-IS were added to the extraction solvent with PCC7002 cells equivalent to 3 mg dry weight. The addition of 5 μL, 10 μL, 20 μL, 50 μL IS were tested for this purpose. Extraction and MS/MS analysis were performed with the method described in the Experimental Section. Then, the absolute values of log_10_ (^12^C/^13^C) were calculated by following the Equation:

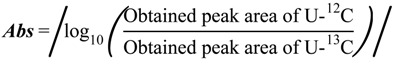
(1)


**Figure 1:**
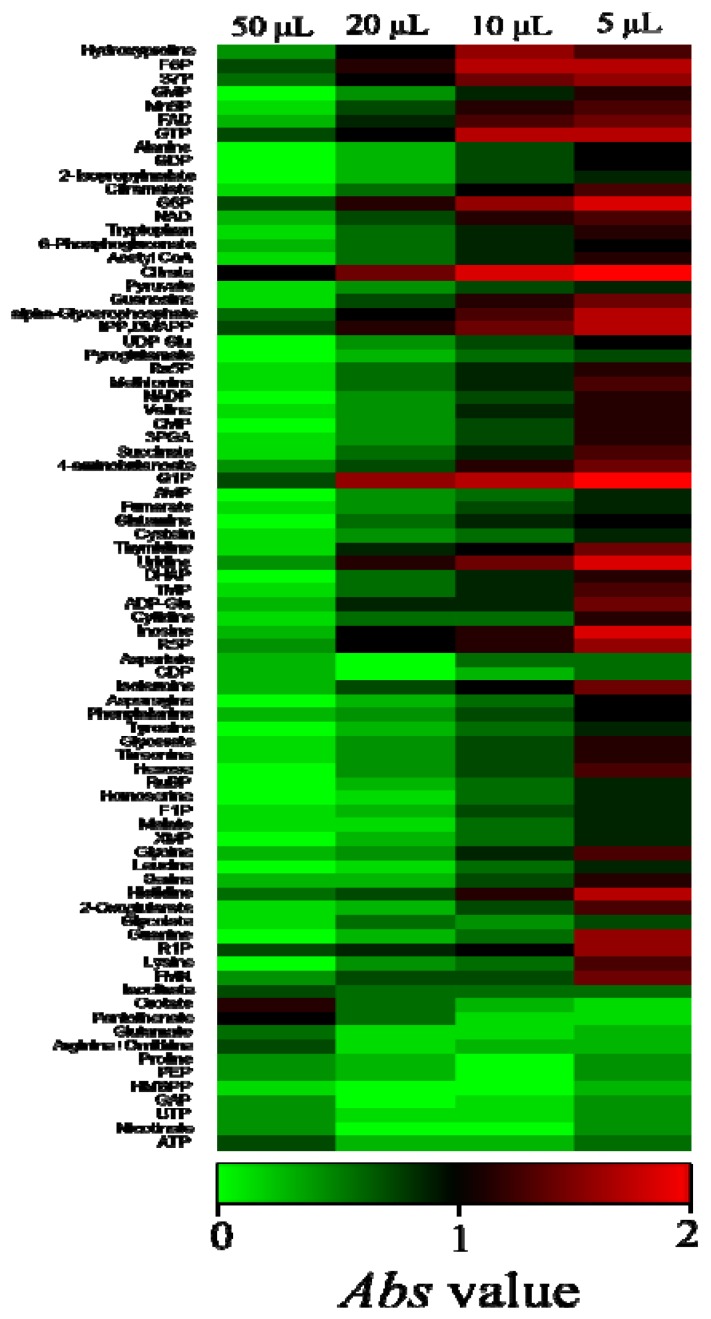
Comparison of area ratios of monoisotopic peaks to their corresponding fully-labeled peaks with different amounts of ^13^C-internal standards. Absolute values of log_10_ (^12^C/^13^C) are represented with a heat map. Abbreviations: 3PGA, 3-phosphoglycerate; Acetyl-CoA, acetyl coenzyme A; ADP, adenosine diphosphate; ADP-Glc, ADP- glucose; AMP, adenosine monophosphate; ATP, adenosine triphosphate; CMP, cytosine monophosphate; CTP, cytosine triphosphate; DHAP, dihydroxyacetone phosphate; Disaccharide-P, disaccharide phosphate; F1P, fructose 1-phosphate; F6P, fructose 6-phosphate; FAD, flavin adenine dinucleotide; FBP, fructose bisphosphate; FMN, flavin mononucleotide; G1P, glucose 1-phosphate; G6P, glucose 6-phosphate; GAP, glyceraldehyde 3-phosphate; GDP, guanosine diphosphate; GMP, guanosine monophosphate; GTP, guanosine triphosphate;HMBPP, 4-Hydroxy-3-methyl-but-2-enyl pyrophosphate; IMP, inosine monophosphate; IPP, isopentenyl pyrophosphate; DMAPP, dimethylallyl pyrophosphate; MEP, 2-C-methylerythritol 4-phosphate; Mn6P, mannose 6-phosphate; NAD, nicotinamide adenine dinucleotide; NADP, nicotinamide adenine dinucleotide phosphate; PEP, phosphoenolpyruvate; R1P, ribose 1-phosphate; R5P, ribose 5-phosphate; Ru5P, ribulose 5-phosphate; RuBP, ribulose bisphosphate; S7P, sedoheptulose 7-phosphate; TMP, thymidine monophosphate; UDP-Glc, UDP-glucose; UMP, uridine monophosphate; UTP, uridine triphosphate; XMP, xanthosine monophosphate.

The *Abs* value represents 10-fold differences between the monoisotopic peak area and fully-labeled peak area. According to the *Abs* value, 50 μL of ^13^C-IS was the most suitable amount to be added in the samples (Figure 1). Thus, we added 50 μL of ^13^C-IS to the extraction solvents in all experiments including the calibration curve construction.

### 2.2. Overview of the Metabolic Differences between Type Strains

**Figure 2:**
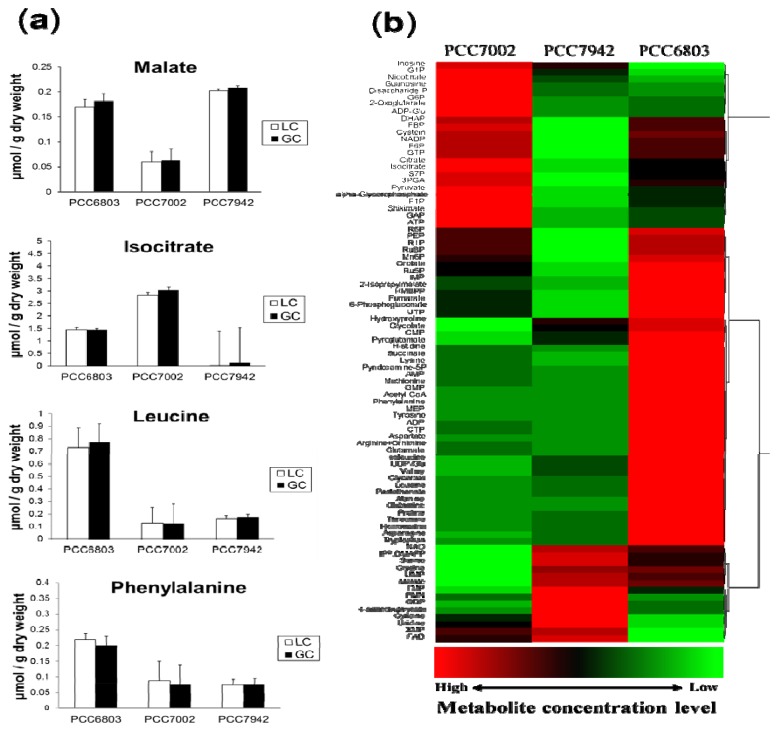
(**a**) Comparison of the absolute concentration values from GC/QqQ-MS and LC/QqQ-MS systems. The bar graphs represent the mean values of triplicate samples and error bars indicate the standard deviations. These metabolites were successfully detected by both systems and resulted in similar values of absolute concentration; (**b**) Comparison of the metabolic profiles of the three cyanobacterial strains, namely, PCC7002, PCC7942 and PCC6803 under photoautotrophic conditions. Z-scored data were hierarchically clustered and the results are represented with a heat map.

Using our targeted metabolic profiling method, the absolute cellular concentrations of 84 metabolites were successfully determined in the three cyanobacterial strains. All metabolite concentrations were standardized by g-dry cell weight (Table 1). Fifteen metabolites (organic acids and amino acids) were analyzed by both GC/QqQ-MS and LC/QqQ-MS systems. Although calculated from different calibration curves, the absolute concentration of the same metabolites derived from two different analytical systems showed similar values and standard deviation (Figure 2a). This result indicates that each analytical system is sufficiently accurate on its own to represent the same data sets. In this study, we selected one data set that has smaller standard deviation.

**Table 1:** Absolute concentrations of each metabolite in μmol/g-drycell weight. S.D. represents standard deviation.

Metabolite	PCC6803		PCC7002		PCC7942	
	Average	S.D.	Average	S.D.	Average	S.D.
2-Isopropylmalate	4.11 × 10 ^−1^	7.75 × 10 ^−2^	1.34 × 10 ^−1^	2.01 × 10 ^−2^	3.22 × 10 ^–2^	1.18 × 10 ^−2^
2-Oxoglutarate	2.94 × 10 ^−1^	7.06 × 10 ^−2^	1.77 × 10^0^	7.23 × 10 ^−1^	1.77 × 10 ^−1^	5.14 × 10 ^−2^
3PGA	1.48 × 10^1^	1.86 × 10^0^	2.02 × 10^1^	4.87 × 10^0^	7.13 × 10^0^	1.18 × 10^0^
4-Aminobutanoate	9.67 × 10 ^−3^	2.09 × 10 ^−3^	9.23 × 10 ^−3^	2.40 × 10 ^−3^	1.42 × 10 ^−2^	1.01 × 10 ^−2^
6-Phosphogluconate	4.66 × 10^0^	8.35 × 10 ^−1^	1.99 × 10^0^	1.11 × 10^0^	1.87 × 10 ^−1^	4.76 × 10 ^−2^
Acetyl CoA	4.43 × 10 ^−1^	4.29 × 10 ^−2^	6.22 × 10 ^−2^	8.02 × 10 ^−3^	6.54 × 10 ^−2^	9.12 × 10 ^−3^
ADP	6.51 × 10^0^	7.58 × 10 ^−1^	2.68 × 10^0^	1.46 × 10 ^−2^	2.38 × 10^0^	6.44 × 10 ^−1^
ADP-Glc	7.00 × 10 ^−2^	4.13 × 10 ^−2^	6.13 × 10 ^−1^	2.66 × 10 ^−1^	3.88 × 10 ^−2^	1.76 × 10 ^−2^
Alanine	4.53 × 10^0^	1.16 × 10^0^	6.50 × 10 ^−1^	1.48 × 10 ^−1^	8.12 × 10 ^−1^	6.74 × 10 ^−1^
α-Glycerophosphate	4.77 × 10 ^−1^	7.50 × 10 ^−2^	9.83 × 10 ^−1^	1.94 × 10 ^−1^	2.22 × 10 ^−1^	3.74 × 10 ^−2^
AMP	1.07 × 10^1^	1.48 × 10^0^	4.56 × 10^0^	4.09 × 10 ^−1^	3.92 × 10^0^	1.09 × 10^0^
Arginine + Ornithine	2.12 × 10 ^1^	5.02 × 10^0^	4.31 × 10^0^	6.72 × 10 ^−1^	3.52 × 10^0^	9.79 × 10 ^−1^
Asparagine	4.34 × 10 ^−1^	8.01 × 10 ^−2^	2.66 × 10 ^−2^	3.97 × 10 ^−3^	8.68 × 10 ^−2^	3.49 × 10 ^−2^
Aspartate	7.14 × 10^0^	1.20 × 10^0^	2.69 × 10^0^	6.09 × 10 ^-1^	2.53 × 10^0^	1.07 × 10^0^
ATP	2.14 × 10^0^	5.58 × 10 ^−1^	3.09 × 10^0^	3.48 × 10 ^−1^	1.90 × 10^0^	8.72 × 10 ^−1^
Citrate	2.16 × 10^0^	7.83 × 10 ^−2^	4.22 × 10^0^	1.43 × 10^0^	3.57 × 10 ^−1^	8.69 × 10 ^−2^
CMP	3.43 × 10 ^−1^	7.71 × 10 ^−2^	2.17 × 10 ^−1^	4.07 × 10 ^−2^	2.71 × 10 ^−1^	3.67 × 10 ^−2^
CTP	5.97 × 10 ^−1^	1.08 × 10 ^-1^	6.52 × 10 ^−2^	9.81 × 10 ^−3^	2.97 × 10 ^−2^	1.13 × 10 ^−2^
Cystein	4.79 × 10 ^−1^	2.30 × 10 ^−1^	5.28 × 10 ^−1^	1.73 × 10 ^−1^	2.32 × 10 ^−1^	1.89 × 10 ^−1^
Cytidine	1.28 × 10 ^−2^	4.16 × 10 ^−3^	2.11 × 10 ^−2^	5.56 × 10 ^−3^	3.40 × 10 ^−2^	7.35 × 10 ^−3^
DHAP	4.30 × 10 ^−1^	7.71 × 10 ^−2^	5.48 × 10 ^−1^	2.91 × 10 ^−1^	1.15 × 10 ^−1^	1.67 × 10 ^−2^
Disaccharide-P	6.03 × 10 ^−4^	3.27 × 10 ^−4^	1.21 × 10 ^−2^	9.63 × 10 ^−3^	1.56 × 10 ^−3^	1.05 × 10 ^−3^
F1P	1.12 × 10 ^−2^	3.71 × 10 ^−3^	1.90 × 10 ^−2^	4.37 × 10 ^−3^	6.99 × 10 ^−3^	1.04 × 10 ^−3^
F6P	4.61 × 10 ^−1^	9.81 × 10 ^−2^	5.19 × 10 ^−1^	2.13 × 10 ^−2^	2.75 × 10 ^−1^	7.97 × 10 ^−2^
FAD	9.98 × 10 ^−2^	3.19 × 10 ^−2^	1.60 × 10 ^−1^	3.38 × 10 ^−2^	1.98 × 10 ^−1^	2.80 × 10 ^−2^
FBP	2.40 × 10 ^−1^	1.07 × 10 ^−1^	3.39 × 10 ^−1^	1.44 × 10 ^−2^	2.08 × 10 ^−2^	1.37 × 10 ^−3^
FMN	4.77 × 10 ^−2^	1.95 × 10 ^−2^	4.81 × 10 ^−2^	7.94 × 10 ^−3^	5.26 × 10 ^−2^	2.18 × 10 ^−2^
Fumarate	1.63 × 10 ^−1^	2.27 × 10 ^−2^	9.08 × 10 ^−2^	9.45 × 10 ^−3^	5.11 × 10 ^−2^	9.58 × 10 ^−3^
G1P	6.91 × 10 ^−2^	1.04 × 10 ^−2^	1.41 × 10 ^−1^	1.45 × 10 ^−2^	9.23 × 10 ^−2^	4.99 × 10 ^−2^
G6P	1.84 × 10^0^	3.10 × 10 ^−1^	4.19 × 10^0^	7.29 × 10 ^−1^	1.57 × 10^0^	3.43 × 10 ^−1^
GAP	2.63 × 10 ^−1^	7.81 × 10 ^−2^	5.59 × 10 ^−1^	2.24 × 10 ^−1^	1.88 × 10 ^−1^	4.91 × 10 ^−2^
GDP	1.38 × 10^0^	1.73 × 10 ^−1^	9.38 × 10 ^−1^	1.77 × 10 ^−^^1^	3.46 × 10^0^	4.42 × 10^0^
Glutamate	2.13 × 10^2^	4.79 × 10^1^	4.25 × 10^1^	6.52 × 10^0^	3.39 × 10^1^	8.11 × 10^0^
Glutamine	3.47 × 10^0^	4.61 × 10 ^−1^	5.14 × 10 ^−1^	1.37 × 10 ^−2^	6.34 × 10 ^−1^	2.12 × 10 ^−1^
Glycerate	2.53 × 10 ^−2^	9.28 × 10 ^−3^	2.06 × 10 ^−2^	5.33 × 10 ^−4^	2.09 × 10 ^−2^	1.93 × 10 ^−2^
Glycine	1.67 × 10 ^1^	4.51 × 10^0^	2.17 × 10^0^	8.78 × 10 ^−1^	1.78 × 10^1^	2.66 × 10^1^
Glycolate	7.51 × 10 ^−2^	2.03 × 10 ^−2^	3.18 × 10 ^−2^	1.47 × 10 ^−2^	5.44 × 10 ^−2^	1.49 × 10 ^−2^
GMP	7.30 × 10 ^−1^	2.05 × 10 ^−1^	3.75 × 10 ^−1^	3.47 × 10 ^−2^	3.77 × 10 ^−1^	6.40 × 10 ^−2^
GTP	8.31 × 10 ^−1^	2.35 × 10 ^−1^	9.61 × 10 ^−1^	1.93 × 10 ^−1^	4.11 × 10 ^−1^	1.31 × 10 ^−1^
Guanosine	7.26 × 10 ^−2^	3.51 × 10 ^−2^	2.91 × 10 ^−1^	1.92 × 10 ^−1^	9.43 × 10 ^−2^	3.71 × 10 ^−2^
Histidine	1.10 × 10 ^−1^	3.46 × 10 ^−2^	7.03 × 10 ^−2^	2.20 × 10 ^−2^	6.45 × 10 ^−2^	2.58 × 10 ^−2^
HMBPP	9.27 × 10 ^−2^	2.66 × 10 ^−2^	5.40 × 10 ^−2^	1.90 × 10 ^−2^	3.32 × 10 ^−2^	4.33 × 10 ^−3^
Homoserine	3.05 × 10 ^−2^	1.16 × 10 ^−2^	1.32 × 10 ^−2^	1.33 × 10 ^−3^	1.51 × 10 ^−2^	3.85 × 10 ^−3^
Hydroxyproline	9.27 × 10 ^−1^	3.05 × 10 ^−1^	4.44 × 10 ^−1^	1.72 × 10 ^−1^	7.65 × 10 ^−1^	5.11 × 10 ^−1^
IMP	4.64 × 10 ^−1^	3.84 × 10 ^−2^	2.48 × 10 ^−1^	9.97 × 10 ^−2^	1.72 × 10 ^−1^	6.00 × 10 ^−2^
Inosine	1.46 × 10 ^−2^	5.54 × 10 ^−3^	9.41 × 10 ^−2^	8.20 × 10 ^−2^	6.80 × 10 ^−2^	2.51 × 10 ^−2^
IPP + DMAPP	1.40 × 10 ^−2^	1.60 × 10 ^−3^	9.64 × 10 ^− 3^	1.79 × 10 ^−3^	1.63 × 10 ^−2^	1.28 × 10 ^−3^
Isocitrate	1.45 × 10^0^	1.25 × 10 ^−1^	3.04 × 10^0^	1.43 × 10^0^	1.09 × 10 ^−1^	4.96 × 10 ^−3^
Isoleucine	3.70 × 10 ^−1^	9.37 × 10 ^−2^	7.14 × 10 ^−2^	1.37 × 10 ^−2^	1.50 × 10 ^−1^	1.16 × 10 ^−1^
Leucine	7.72 × 10 ^−1^	1.55 × 10 ^−1^	1.26 × 10 ^−1^	2.32 × 10 ^−2^	1.76 × 10 ^−1^	1.33 × 10 ^−1^
Lysine	1.57 × 10 ^−1^	4.40 × 10 ^−2^	1.02 × 10 ^−1^	1.11 × 10 ^−2^	9.32 × 10 ^−2^	3.62 × 10 ^−2^
Malate	1.82 × 10 ^−1^	2.24 × 10 ^−2^	6.36 × 10 ^−2^	4.72 × 10 ^−3^	2.08 × 10 ^−1^	7.92 × 10 ^−2^
MEP	8.38 × 10 ^−2^	1.64 × 10 ^−2^	3.94 × 10 ^−2^	9.17 × 10 ^−3^	3.83 × 10 ^−2^	8.08 × 10 ^−3^
Methionine	6.13 × 10 ^−1^	1.10 × 10 ^−1^	2.67 × 10 ^−1^	4.40 × 10 ^−2^	2.30 × 10 ^−1^	4.51 × 10 ^−2^
Mn6P	2.92 × 10 ^−1^	4.19 × 10 ^−2^	2.53 × 10 ^−1^	7.48 × 10 ^−2^	2.02 × 10 ^−1^	3.06 × 10 ^−2^
NAD	5.14 × 10 ^−1^	1.26 × 10 ^−1^	3.84 × 10 ^−1^	2.97 × 10 ^−2^	5.64 × 10 ^−1^	1.04 × 10 ^−1^
NADP	6.14 × 10 ^−1^	1.26 × 10 ^−1^	6.82 × 10 ^−1^	1.38 × 10 ^−1^	3.79 × 10 ^−1^	8.28 × 10 ^−2^
Nicotinate	3.56 × 10 ^−4^	7.66 × 10 ^−5^	7.72 × 10 ^−4^	3.27 × 10 ^−4^	4.62 × 10 ^−4^	1.86 × 10 ^−4^
Orotate	1.03 × 10 ^−2^	1.58 × 10 ^−3^	5.97 × 10 ^−3^	2.34 × 10 ^−3^	1.75 × 10 ^−3^	7.23 × 10 ^−4^
Pantothenate	4.91 × 10 ^−3^	1.02 × 10 ^−3^	2.13 × 10 ^−4^	3.22 × 10 ^−5^	4.56 × 10 ^−4^	2.59 × 10 ^−4^
PEP	2.51 × 10^0^	7.59 × 10 ^−1^	2.27 × 10 ^0^	7.18 × 10 ^−1^	1.47 × 10^0^	2.45 × 10 ^−1^
Phenylalanine	1.99 × 10 ^−1^	6.09 × 10 ^−2^	7.73 × 10 ^−2^	1.86 × 10 ^−2^	7.63 × 10 ^−2^	4.77 × 10 ^−2^
Proline	9.14 × 10 ^−1^	1.76 × 10 ^−1^	5.06 × 10 ^−2^	9.05 × 10 ^−3^	1.38 × 10 ^−1^	1.23 × 10 ^−1^
Pyridoxamine-5P	4.58 × 10 ^−2^	5.05 × 10 ^−3^	3.32 × 10 ^−2^	2.92 × 10 ^−3^	3.17 × 10 ^−2^	3.82 × 10 ^−3^
Pyroglutamate	2.81 × 10^0^	7.22 × 10 ^−1^	5.95 × 10 ^−1^	8.06 × 10 ^−2^	1.51 × 10^0^	1.41 × 10^0^
Pyruvate	1.05 × 10^1^	1.38 × 10^0^	1.81 × 10 ^1^	4.20 × 10 ^0^	5.76 × 10^0^	1.12 × 10^0^
R1P	3.62 × 10 ^−3^	7.87 × 10 ^−4^	2.90 × 10 ^−3^	1.36 × 10 ^−3^	9.68 × 10 ^−4^	2.08 × 10 ^−4^
R5P	1.95 × 10 ^−1^	6.88 × 10 ^−2^	1.52 × 10 ^−1^	6.33 × 10 ^−2^	5.82 × 10 ^−2^	8.74 × 10 ^−3^
Ru5P	4.04 × 10 ^−1^	6.01 × 10 ^−2^	2.19 × 10 ^−1^	4.25 × 10 ^−2^	4.48 × 10 ^−2^	5.98 × 10 ^−3^
RuBP	2.92 × 10 ^−1^	5.35 × 10 ^−2^	2.37 × 10 ^−1^	5.29 × 10 ^−2^	8.39 × 10 ^−2^	2.74 × 10 ^−2^
S7P	3.57 × 10^0^	1.10 × 10^0^	5.60 × 10 ^0^	2.50 × 10 ^0^	1.28 × 10^0^	4.41 × 10 ^−1^
Serine	7.12 × 10 ^−1^	2.63 × 10 ^−1^	3.11 × 10 ^−1^	9.71 × 10 ^−2^	9.12 × 10 ^−1^	1.01 × 10^0^
Shikimate	1.32 × 10 ^−3^	5.41 × 10 ^−4^	1.66 × 10 ^−3^	1.13 × 10 ^−3^	1.19 × 10 ^−3^	7.67 × 10 ^−4^
Succinate	3.23 × 10 ^−1^	7.42 × 10 ^−2^	2.15 × 10 ^−1^	6.81 × 10 ^−2^	1.97 × 10 ^−1^	4.21 × 10 ^−2^
Threonine	8.39 × 10 ^−1^	1.99 × 10 ^−1^	2.95 × 10 ^−1^	8.09 × 10 ^−2^	3.52 × 10 ^–1^	2.68 × 10 ^−1^
TMP	2.71 × 10 ^−2^	6.61 × 10 ^−3^	2.23 × 10 ^−2^	6.80 × 10 ^−3^	3.57 × 10 ^−2^	2.71 × 10 ^−3^
Tryptophan	2.19 × 10 ^−1^	4.26 × 10 ^−2^	6.95 × 10 ^−2^	7.88 × 10 ^−3^	8.85 × 10 ^−2^	1.18 × 10 ^−2^
Tyrosine	3.04 × 10^0^	6.83 × 10 ^−1^	6.09 × 10 ^−1^	6.31 × 10 ^−2^	5.62 × 10 ^−1^	2.60 × 10 ^−1^
UDP-Glc	2.24 × 10^0^	2.22 × 10 ^−1^	6.98 × 10 ^−1^	7.48 × 10 ^−2^	1.05 × 10^0^	1.97 × 10 ^−1^
UMP	7.44 × 10 ^−1^	1.52 × 10 ^−1^	6.05 × 10 ^−1^	2.43 × 10 ^−1^	7.79 × 10 ^−1^	1.58 × 10 ^−1^
Uridine	2.16 × 10 ^−2^	9.04 × 10 ^−3^	5.21 × 10 ^−2^	3.01 × 10 ^−2^	8.93 × 10 ^−2^	2.92 × 10 ^−2^
UTP	5.54 × 10 ^−1^	1.49 × 10 ^−1^	3.19 × 10 ^−1^	4.17 × 10 ^−2^	1.75 × 10 ^−1^	4.96 × 10 ^−2^
Valine	9.81 × 10 ^−1^	2.00 × 10 ^−1^	2.09 × 10 ^−1^	3.94 × 10 ^−2^	3.60 × 10 ^−1^	2.13 × 10 ^−1^
XMP	4.73 × 10 ^−2^	1.37 × 10 ^−2^	8.66 × 10 ^−2^	3.06 × 10 ^−2^	1.01 × 10 ^−1^	2.73 × 10 ^−2^

For biological production, high proliferative ability and capability for genetic modification are important (Table 2). The cyanobacterial strains used in this study are genetically well-studied and the whole genome information is available. Therefore, they have potential to be attractive host strain candidates for metabolic engineering. From our results, the metabolic profile of the PCC6803 strain clearly indicated that most amino acids were present in higher amounts and this tendency was also observed in the other *Synechocystis* strain: *Synechocystis* sp. PCC6714 (Supplementary Figure 1). Although it is known that the two types of *Synechocystis* strains, PCC6803 and PCC6714, have several physiological differences such as salinity response, they are closely related from the viewpoint of their genome (16S rDNA identity 99.4%) [26]. Nevertheless, the metabolic profiles of these two strains are clearly different from each other in terms of several metabolites (Supplementary Figure 1). These results confirm that metabolomics has the ability for high-resolution phenotypic analysis that can differentiate genetically similar strains, which can be informative when choosing the appropriate host for biological production. On the other hand, some intermediates of the primary carbon metabolic pathway such as Calvin and TCA cycles were notably accumulated only in PCC7002 (Figure 2b). It was previously reported that the TCA cycle activity exhibited a strong correlation with the growth rate of *Saccharomyces cerevisiae* [27] and this phenomenon is expected to occur in other aerobic microorganisms. However, we could not find any relationship between the level of TCA cycle intermediates and the growth rate in three cyanobacterial strains. This might be due to the “unusual” TCA cycle of cyanobacteria [28] and their distinct energy acquisition strategy derived from the photosystem. Although a novel insight for cyanobacterial cyclic TCA cycle was reported wherein succinic semialdehyde was suggested to be a new intermediate of TCA cycle [29], the metabolite could not be detected in either strain. It is probable that succinic semialdehyde is not accumulated under constant light condition, although this assumption needs confirmation.

**Table 2:** Phenotypic characterization of the strains used in this study.

Strain	Growth Rate (h^−1^) ^1^	Genetic Modification	Utilizable Organic Substrate
*Synechocystis* sp. PCC6803	0.107 ± 0.003	[30]	Glucose ^2^
*Synechococcus* sp. PCC7002	0.165 ± 0.002	[6]	Glucose, Glycerol
*Synechococcus elongatus* PCC7942	0.123 ± 0.002	[31]	None

^1^ The growth rate is measured and expressed as the mean ± S.D. (*n* = 3); ^2^ In case of the glucose-tolerant strain.

### 2.3. Characterization of Each Strain by Molar-Based Metabolite Distribution and AEC Values

**Figure 3:**
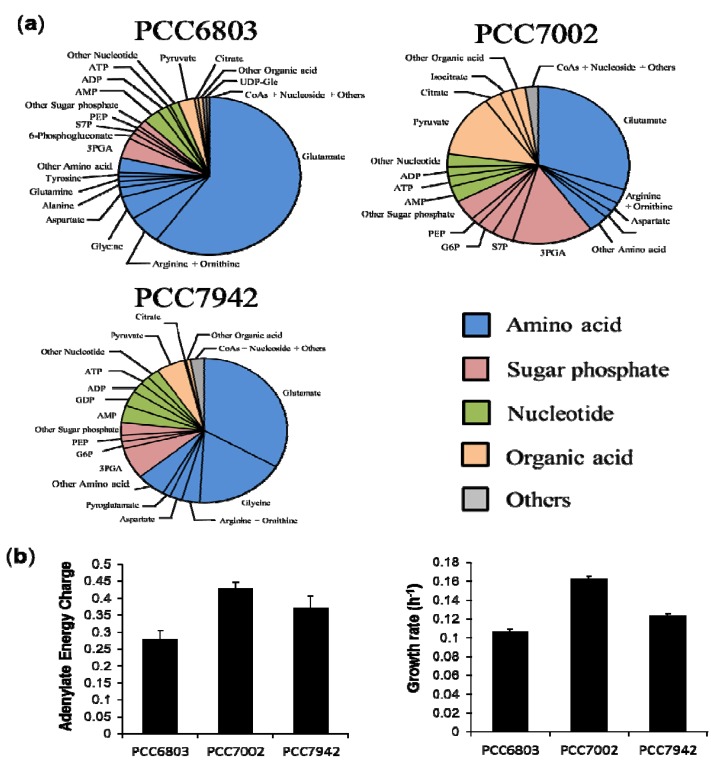
(**a**) Composition of the measured metabolome in three cyanobacteria. The pie graph shows the distributions of different metabolites in mid-exponential phase; (**b**) Comparison of adenylate energy charge and growth rate in each strain. The bar graphs indicate the mean values of triplicates and error bars indicate the standard deviation.

The absolute cellular concentrations of 84 metabolites are described in the pie graph of each strain (Figure 3a). Glutamate was the most abundant metabolite in all strains, especially in *Synechocystis* strains; occupying approximately 60% of the total detected metabolites. In these graphs, five metabolite groups were classified namely: amino acids, sugar phosphates, nucleotides, organic acids and others based on their chemical properties or roles in metabolism. Among the highly observed metabolites in the cyanobacterial strains, glutamate, arginine and ornithine are related to nitrogen metabolism [32], 3PGA is a product of carbon fixation reaction, three adenylate nucleotides work as energy currencies, and pyruvate is a final product of glycolysis. These compounds play important roles in energy acquisition or nutrient storage. In spite of the similar lineup of metabolites, the molar-based group distribution of PCC7002 was significantly different from the others. In order to minimize the effects of different culture conditions on metabolome, we cultivated marine cyanobacterium PCC7002 at the same temperature and light conditions with the other two strains except for modified BG-11 medium. Although the temperature in our condition is relatively low compared to the optimal condition, there is no significant difference in the doubling time of PCC7002 between our condition (4.26 ± 0.06 h) and the optimal condition (4 ± 0.3 h) [33]. However, it is necessary to point out that low temperature has been shown to cause nitrogen limitation in PCC7002 [33], which probably explained the notable accumulation of 2-oxoglutarate and low levels of glutamate and other related amino acids in PCC7002 under our condition. The adenine nucleotides ATP, ADP and AMP stoichiometrically couple with global metabolic pathways of a living cell in which metabolically available energy is momentarily stored in the form of energy-rich phosphate bonds. Therefore, the intracellular adenylate energy condition is expected to reflect phenotypic or metabolic differences and give an informative guide for elucidation of metabolism in various strains of cyanobacteria. To evaluate the intracellular energy condition, we calculated the adenylate energy charge (AEC) in terms of absolute concentrations using the Equation

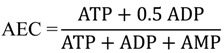
(2)


The AEC value has been proven to be a metabolic regulatory parameter for various enzymatic reactions and their related pathways *in vivo* [34,35]. The lowest AEC value was observed in *Synechocystis*. In contrast, PCC7002 had the highest value among the three strains (Figure 3b) although the AEC values (with a mean of 0.359) were relatively low compared with those of non-photosynthetic microorganisms, for example, *Escherichia coli* (0.90–094), *Saccharomyces cerevisiae* (0.84–0.93) [36]. Based on the AEC values and metabolite distribution data, there seems to be a relationship between th AEC value and accumulation of phospho-related metabolites. More interestingly, we found very similar tendencies between AEC values and the growth rate of mid-exponential phase in the three investigated strains and as well as in PCC6714 (Supplementary Figure 2). The growth rate of each strain was estimated by its growth curves during exponential growth phase (Supplementary Figure 3). Since the Pearson correlation coefficient using mean values was 0.906, AEC values were strongly correlated with growth rate, but not with the absolute concentration of ATP. This result indicates that cyanobacterial growth during mid-log phase is directly or indirectly dominated by cellular energy states. Although a well-correlated relationship between AEC and growth in *Escherichia coli* has been reported in previous research [36], we did not expect this result because cyanobacteria have a completely different energy strategy from aerobionts such as *E.coli*. Cyanobacteria mainly obtain ATP from photosynthesis but most aerobionts get ATP from cellular respiration. Since ATP has been known as an activator of ribulosebisphosphate carboxylase/oxygenase which is a key enzyme of calvin-benson cycle [37], cellular energy state should have affected growth by a different mechanism from aerobionts. Although an approach to enhance the proliferative ability—which is important for cyanobacterial biological production—by optimizing the upstream cultivation parameters has been described [38], there is hitherto no approach based on cellular energy state. Our result demonstrated a new possibility of improving cyanobacterial growth from a completely different aspect. On the other hand, the information on organic carbon distribution is likely to play an important role in selecting appropriate strains for specific biochemical production because several metabolites can be precursors of important bioproducts.

### 2.4. Overview and Prospect for Cyanobacterial Biological Production

**Figure 4:**
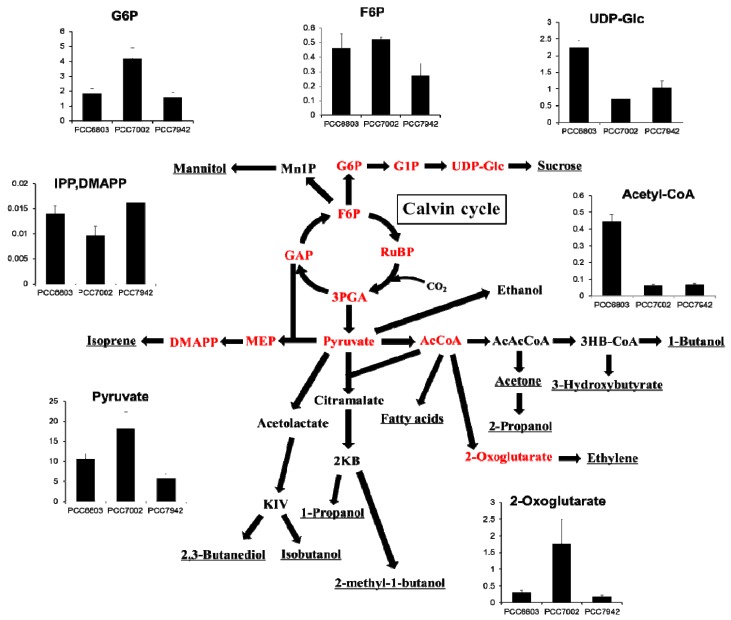
Overview of the modified metabolic pathways related to previously reported cyanobacterial biological production. The targeted products are underlined while the metabolites in red can be quantitated by our method. The vertical axes of the bar graphs show the intercellular concentrations in μmol/g-dry cell weight of each metabolite (mean ± S.D., *n* = 3).

As described previously, there are many challenges in cyanobacterial biological production. Most of these researches in cyanobacteria are using genetically modified strains with introduced exogenous genes because the wild type strain has low or no ability to produce valuable biofuels such as alcohols or some kinds of carbohydrates. However, the end products are derived from naturally occurring precursors. Assuming a simple Michaelis-Menten enzymatic reaction without the allosteric effect, the reaction rate unambiguously depends on the molar concentration of the substrate. Therefore, monitoring such kind of precursors and determining the metabolic rate-limiting points are important and essential considerations in metabolic engineering even when using wild type strains. In order to visualize the previous reports of cyanobacterial production, we summarized the modified metabolic pathways, which described the relationship between primary metabolites and various targeted products (Figure 4). This network clearly showed that most targeted products were closely linked to pyruvate or acetyl-CoA, and the level of these two metabolites vary significantly among the three cyanobacterial type strains. In addition, the ratios of pyruvate/acetyl-CoA are commonly high but different among the strains. In the case of PCC7002, the amount of pyruvate was approximately 300-fold relative to that of acetyl-CoA. The reaction from pyruvate to acetyl-CoA, which is highly regulated by complex cell mechanisms [39], might be the limiting step in various metabolic pathways. For further improvement of cyanobacterial biological production through acetyl-CoA, a better alternative pathway which is independent from pyruvate dehydrogenase would be preferable. For example, non-oxidative glycolysis pathway via phosphoketolases seems to be one of the possibilities for avoiding this limiting step [40].

## 3. Experimental Section

### 3.1. Strain and Cultivation

PCC7942 and glucose tolerant mutant of PCC6803 were grown in BG-11 liquid medium (1.5 g/L NaNO_3_, 0.027 g/L CaCl_2_, 0.006 g/L ferric ammonium citrate, 0.001 g/L Na_2_EDTA 2H_2_O, 0.039 g/L K_2_HPO_4_, 0.075 g/L MgSO_4_ 7H_2_O, 0.020 g/L Na_2_CO_3_, 0.006 g/L citrate, 2.860 mg/L H_3_BO_3_, 1.810 mg/L MnCl_2_ 4H_2_O, 0.220 mg/L ZnSO_4_ 7H_2_O, 0.390 mg/L Na_2_MoO_4_ 2H_2_O, 0.080 mg/L CuSO_4_ 5H_2_O, and 0.050 mg/L Co(NO_3_)_2_ 6H_2_O) supplemented with 20 mm HEPES which was used as a basic medium for freshwater Cyanobacteria. PCC7002, a euryhaline cyanobacterium, was grown in modified BG-11 medium supplemented with 20 μg/L cyanocobalamin and 28.8 g/L artificial seawater (Marine Art SF-1; Tomita Pharmaceutical Co., Ltd., Tokushima) instead of ddH_2_O. All cultivations were carried out in clear test tubes (30 × 200 mm; Pyrex^®^, IWAKI, Japan) containing 50 mL of modified BG-11 medium under standard aerobic incubation and constant light exposure. Cells were grown at 30 °C under fluorescent light (85–90 μmol/m^2^ s) and 1% carbon dioxide-air at approximately 80 mL/min. Growth and cell density were determined by measuring the optical density at 730 nm (OD_730_) using a spectrophotometer (GeneQuant 100, GE Healthcare, Piscataway, NJ, USA). Cell density was determined as the dry cell weight in the medium at OD_730_ = 1. Each cold stock was inoculated into 50 mL fresh-water or sea-water BG-11 medium as a pre-culture until OD_730_ = 1. Cultures were transferred to 50 mL of each BG-11 medium with an initial OD_730_ equal to 0.01 and incubated until it reaches an OD_730_ equal to 1. The pre-culture and main culture were incubated in a photosynthesis cultivation system described above. For the main culture, three different tubes for each strain were prepared as biological replicates. At OD_730_ = 1, all three cyanobacteria were in mid-log growth phase in these cultivation system. Some features of the three strains are summarized in Table 2. The information on utilizable organic substrate was obtained from [41].

### 3.2. Sampling Procedure for Metabolic Profiling Analysis

Cell sampling was performed according to a previously reported method [42], with minor modifications. Briefly, cyanobacterial cells equivalent to 3 mg dry weight were removed from cultivation tubes and filtered using 0.2 μm pore size Omnipore filter disks (Millipore, MA, USA). After washing with pre-chilled 70 mm ammonium bicarbonate, cells retained on the filters were immediately placed on an aluminum plate pre-cooled with liquid nitrogen. This step allowed the removal of the culture and immediate quenching of cyanobacterial metabolic activities (<15 sec in total). Subsequently, filters were put into 15 mL centrifuge tubes and stored at −80 °C.

### 3.3. Metabolite Extraction and Sample Preparation

Three milliliters of extraction solvent, which consisted of methanol/water/chloroform (2.5:1:1) was added in each 15 mL centrifuge tube with filtered samples, then ^13^C-internal standard was added before vortexing for 30 sec, and incubation in liquid nitrogen for 2 min. The samples were then allowed to thaw at −30 °C and sonicated for 2 min. This cycle of cell lysis in liquid nitrogen with sonication was repeated three times. This process enables the extraction of intracellular metabolites at low temperatures, and prevents the decomposition of the metabolites. To precipitate proteins and cell pellets, the samples were centrifuged at 16,000 × *g* for 30 min at 4 °C. The resulting 2 mL supernatant was divided equally into two 2 mL-microfuge tubes and mixed with 200 µL of ultrapure water. Centrifugation was performed to separate the nonpolar phase. Approximately 2 mL aliquot from the polar phase was concentrated using a centrifugal concentrator (VC-36S, TAITEC Co., Tokyo, Japan) for 2 h and subsequently, to remove concentrated proteins, centrifuged at 16,000 × *g* for 30 min at 4 °C. The supernatant was reevaporated to a final volume of approximately 100 μL. Twenty μL of sample was collected for IP-LC/QqQ-MS analysis, and for GC/QqQ-MS analysis, 50 μL of sample was freeze dried and derivatized by methoxyamine hydrochloride (Sigma Aldrich, St. Louis, MO, USA) in pyridine (40 µL, 20 mg mL^−^^1^) at 30 °C for 90 min, followed by 40 µL of N-methyl-N-(*tert.*-butyldimethylsilyl) trifluoroacetamide (MTBSTFA) with 1% *tert.*-butyldimethylsilyl chloride (TBDMSCl) (Thermo Scientific, Hudson, NH, USA) at 37 °C for 30 min.

### 3.4. Preparation of ^13^C-Internal Standard

In this study, U-^13^C cyanobacterial cell extract was used as a common internal standard. This extract was derived from two type strains; PCC7942 and PCC6803. Both cyanobacteria were cultivated in 100 mL ^13^C-modified BG-11 (300 mL screw-capped flask) under continuous irradiation with 90 μmol of white light photons m^−2^ s^−1^ (whole-day illumination) with 100 rpm agitation at 30 °C. ^13^C-modified BG-11 initially contained 50 mm NaH^13^CO_3_ (>98 atom% ^13^C, Isotec, Inc., Miamisburg, OH, USA) instead of Na_2_CO_3_ as a carbon source. Fifty mm NaH^13^CO_3_ was added every day until collection. The initial OD_730_ was 0.01, and after three days of cultivation, the samples were collected and extracted using the method described above. Two individual cell extracts were integrated, and the ratio of U-^13^C to U-^13^C+U-^12^C peak area was checked by both system (Supplementary Figure 2) and this sample was used as a ^13^C-internal standard.

### 3.5. GC/QqQ-MS and IP-LC/QqQ-MS Analysis

The GC/QqQ-MS system was an Agilent 7890A series GC system coupled with an Agilent 7000B QqQ-MS (Agilent Technologies, Inc., USA). The column was an InertCap 5MS/Sil (30 m × 0.25 mm × 0.25 µm) (GL Sciences, Tokyo, Japan). The front inlet temperature was 230 °C. The helium gas flow rate through the column was 1 mL/min. The column temperature was held at 80 °C for 2 min isothermally and then raised by 15 °C/min to 330 °C, and held there isothermally for 2 min. The transfer line and the ion source temperature were 250 and 230 °C, respectively. The nitrogen flow rate was 1.5 mL/min as a collision gas. A total of 62 transitions for 31 metabolites (monoisotopic and U-^13^C) were constructed for cyanobacterial analysis. The peaks of each target metabolite were identified by comparison of the chromatographic shapes and retention times with that of the corresponding standards and areas were determined by Agilent Mass Hunter quantitative software version B.04.00 (Agilent).

LC/QqQ-MS analysis was done using the Shimadzu Nexera UHPLC system coupled with LCMS 8030 Plus (Shimadzu Corp., Kyoto, Japan). The column was a PE capped CERI L-column 2 ODS (150 mm × 2.1 mm, particle size 3 μm, Chemicals Evaluation and Research Institute, Tokyo, Japan). The conditions were as follows: mobile phase, 10 mm tributylamine and 15 mm acetic acid in water (A) and methanol (B); flow rate, 0.3 mL/min; gradient curve, 0% B at 0–1, 15% B at 2–4, 50% B at 9, 55% B at 11.5, 100% B at 13–13.5 and 0% B at 13.5–18 min; injection volume, 3 μL; and column oven temperature, 45 °C. The mode of mass analysis was negative ion mode. The probe position was +1.5 mm, the desolvation line temperature was 250 °C, the nebulizer gas flow was 2 L/min, the drying gas flow was 15 L/min, and the heat block temperature was 400 °C. The other MS parameters were determined by auto-tuning. In this analysis, we used the scheduled MRM mode.

A total of 110 metabolites from 220 MRM transitions (monoisotopic and U-^13^C) were targeted in this system. The LC/QqQ-MS system and an ion-pair solvent are often used in widely targeted metabolomics studies [23,43]. The peaks of each target metabolite were identified by comparison of its chromatographic shapes and retention times with that of corresponding standards and those areas were determined by LabSolutions version 5.60 (Shimadzu, Kyoto, Japan).

### 3.6. Determine the Absolute Concentration

Absolute quantitation of the intracellular metabolite levels was performed by using ^12^C/^13^C ratio-based calibration curves. To make calibration curves for each metabolite, predetermined amounts of unlabeled standard mixtures were directly added to separate 15 mL centrifuge tubes containing 3 mL of extraction solvent with the same amount of labeled cell extract as internal standard. Then, the unlabeled standard mixtures and labeled cell extracts were extracted and prepared in the same manner as the cyanobacterial samples (See *Metabolite Extraction and Sample Preparation*).

**Figure 5:**
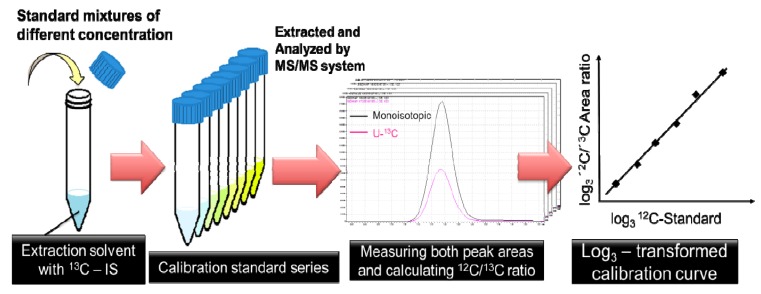
The workflow of making calibration curves. Predetermined amounts of standards were directly added to centrifuging tubes containing 3 mL of extraction solvent with ^13^C-IS. The peak areas of the U-^13^C and monoisotopic (U-^12^C) metabolites were measured by MS/MS. ^12^C/^13^C ratios were plotted against their corresponding known amounts of naturally labeled standards on log_3_-log_3_ transformed liner calibration curves.

The peak areas of the U-^13^C and monoisotopic (U-^12^C) metabolites were measured by LC-ESI-MS/MS and GC-EI-MS/MS. ^12^C/^13^C ratios were calculated and plotted against their corresponding known amounts of naturally labeled standards. We made log_3_-log_3_ transformed liner calibration curves based on at least five points, and absolute concentrations in experimental samples were computed from the intercepts and slopes of the calibration curves (Supplementary Figure 4). The points on calibration curves correspond to 0.05, 0.15, 0.45, 1.35, 4.05, 12.15, 36.45, 109.35 nmol per tube, respectively (Figure 5).

## 4. Conclusions

Triple quadrupole mass spectrometry-based target metabolic profiling method for cyanobacteria was developed. By using ^13^C-labeled cell extracts as internal standards, we successfully determined the absolute concentrations of 84 metabolites in three different cyanobacterial type strains used in this study. The overview of respective metabolic profiling results showed that *Synechocystis* strains had a different tendency from *Synechococcus* strains in terms of the distribution of some metabolites such as amino acids and sugar phosphates. Moreover, the growth rate of the three investigated strains seemed to be correlated with adenylate energy charge value rather than TCA cycle activity. This might indicate that the cyanobacterial TCA cycle does not have a role in supplying energies for the microorganism’s cellular metabolism. Finally, we suggested a candidate for the limiting step in cyanobacterial biological production under photoautotrophic conditions. The data obtained and insights revealed in this study are expected to contribute to cyanobacterial physiological and industrial researches.

## Supplementary Files

Supplementary File 1 Supplementary Information (PDF, 1480 KB)
