# Supplementary material for: Molar-Based Targeted Metabolic Profiling of Cyanobacterial Strains with Potential for Biological Production
Source: Metabolites. 2014 Jun 20;4(2):499–516. doi: 10.3390/metabo4020499 (PMC4101518; doi:10.3390/metabo4020499)
Supplement: Supplementary File 1 — Supplementary Information (PDF, 1480 KB) [file metabolites-04-00499-s001.pdf]

Supplementary Information

Figure 1. Comparison of the metabolic profiles of the three cyanobacterial strains plus additional Synechocystis strain, namely, PCC7002, PCC7942, PCC6803 and PCC6714 under photoautotrophic conditions. Z-scored data were hierarchically-clustered and the results are represented with a heat map.

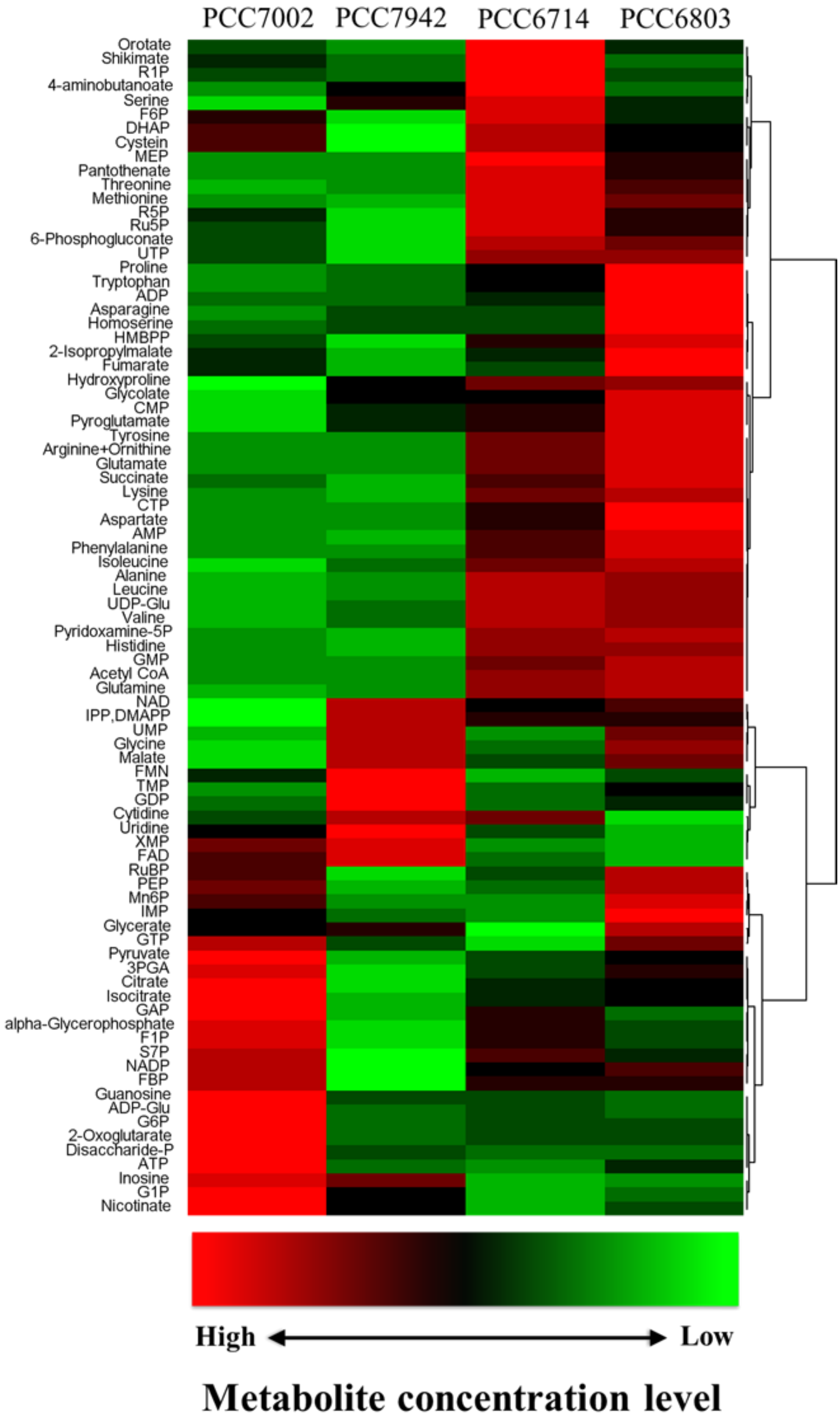

Figure 2. Comparison of adenylate energy charge and growth rate in four strains. The bar graphs indicate the mean values of triplicates and error bars indicate the standard deviation. Pearson correlation coefficient by using all four mean values is 0.906.

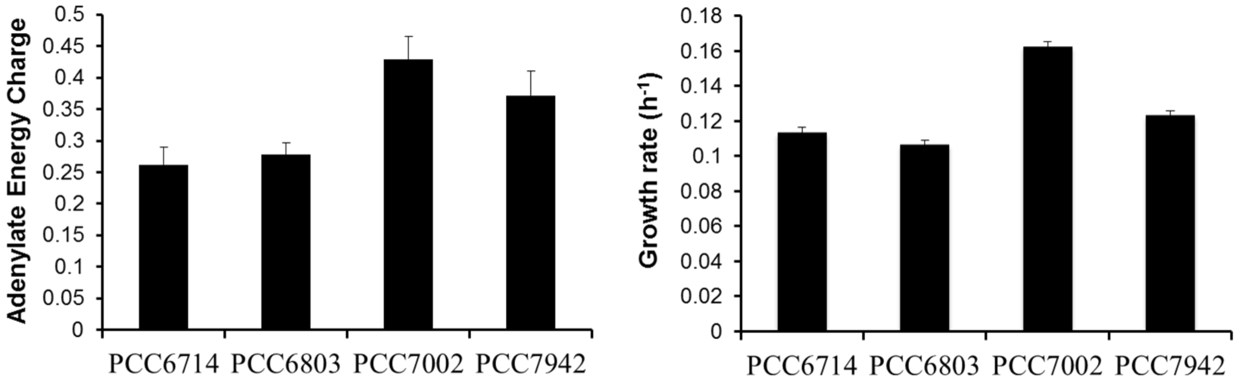

Table 1. The quantitation result of *Synechocystis* sp. PCC6714. The values indicate the mean of triplicates in μmol/g-drycell weight and S.D. represents standard deviation.

**Metabolite**	Average	S.D.	**Metabolite**	Average	S.D.
2-Isopropylmalate	1.48E-01	2.47E-02	Glutamine	3.33E+00	3.12E-01
2-Oxoglutarate	3.42E-01	8.26E-02	Glycerate	1.37E-02	2.14E-03
3PGA	1.12E+01	1.82E+00	Glycine	7.05E+00	6.33E-01
4-Aminobutanoate	2.41E-02	5.34E-03	Glycolate	5.29E-02	1.15E-02
6-Phosphogluconate	5.64E+00	6.73E-01	GMP	6.63E-01	1.09E-01
Acetyl CoA	4.13E-01	5.08E-02	GTP	8.51E-02	1.47E-01
ADP	3.10E+00	2.36E-01	Guanosine	1.12E-01	2.56E-02
ADP-Glc	1.13E-01	7.02E-02	Histidine	1.08E-01	3.14E-03
Alanine	4.79E+00	1.02E+00	HMBPP	6.72E-02	2.50E-02
α-Glycerophosphate	6.24E-01	7.70E-02	Homoserine	1.59E-02	2.14E-03
AMP	7.89E+00	7.38E-01	Hydroxyproline	8.77E-01	1.73E-01
Arginine + Ornithine	1.59E+01	3.77E+00	IMP	1.18E-01	5.08E-02
Asparagine	1.04E-01	1.18E-02	Inosine	9.14E-03	1.50E-03
Aspartate	4.65E+00	7.86E-01	IPP + DMAPP	1.39E-02	3.60E-03
ATP	1.80E+00	3.12E-01	Isocitrate	1.11E+00	4.67E-02
Citrate	1.90E+00	2.24E-01	Isoleucine	3.02E-01	4.86E-02
CMP	2.85E-01	4.39E-02	Leucine	8.26E-01	1.48E-01
CTP	3.11E-01	8.63E-02	Lysine	1.42E-01	2.47E-02
Cystein	6.22E-01	3.41E-01	Malate	1.20E-01	1.52E-02
Cytidine	2.91E-02	4.83E-03	MEP	1.36E-01	2.95E-02
DHAP	6.47E-01	9.46E-02	Methionine	7.57E-01	1.19E-01
Disaccharide-P	0.00E+00	0.00E+00	Mn6P	2.03E-01	1.62E-01
F1P	1.40E-02	2.80E-03	NAD	4.85E-01	3.41E-02
F6P	6.89E-01	5.95E-02	NADP	5.73E-01	8.69E-02

FAD	1.13E-01	8.14E-03	Nicotinate	1.99E-04	2.03E-05
FBP	2.50E-01	8.28E-02	Orotate	2.92E-02	7.73E-03
FMN	4.61E-02	1.10E-02	Pantothenate	9.20E-03	3.24E-03
Fumarate	7.72E-02	2.92E-03	PEP	1.71E+00	2.11E-01
G1P	5.26E-02	5.90E-03	Phenylalanine	1.48E-01	2.87E-02
G6P	1.91E+00	5.04E-01	Proline	3.30E-01	5.23E-02
GAP	3.84E-01	4.50E-02	Pyridoxamine-5P	4.53E-02	6.92E-03
GDP	8.25E-01	1.30E-01	Pyroglutamate	1.99E+00	3.63E-01
Glutamate	1.59E+02	3.69E+01	Pyruvate	8.02E+00	9.53E-01
R5P	2.80E-01	6.46E-02	R1P	2.04E-02	2.24E-03
Ru5P	2.04E-02	1.31E-01	Tryptophan	2.24E-03	2.11E-02
RuBP	2.80E-01	2.27E+00	Tyrosine	6.46E-02	4.19E-01
S7P	6.46E-01	2.31E+00	UDP-Glc	5.14E-02	3.78E-01
Serine	1.57E-01	6.20E-01	UMP	2.76E-02	1.26E-01
Shikimate	4.57E+00	4.15E-02	Uridine	5.10E-01	8.48E-03
Succinate	1.35E+00	5.31E-01	UTP	1.00E-01	2.83E-02
Threonine	3.75E-03	1.08E+00	Valine	1.99E-03	1.83E-01
TMP	2.83E-01	5.02E-02	XMP	3.35E-02	8.01E-03

Figure 3. Growth curves in *Synechococcus elongatus* PCC7942, *Synechococcus* sp. PCC7002, *Synechocystis* sp. PCC6803 and *Synechocystis* sp. PCC6714 at 30 °C under fluorescent light (85-90 μmol/m^2·s) and 1% CO_2-Air. The bar graphs indicate the mean values of triplicates and error bars indicate the standard deviation. Growth was monitored by the optical density at 730 nm.

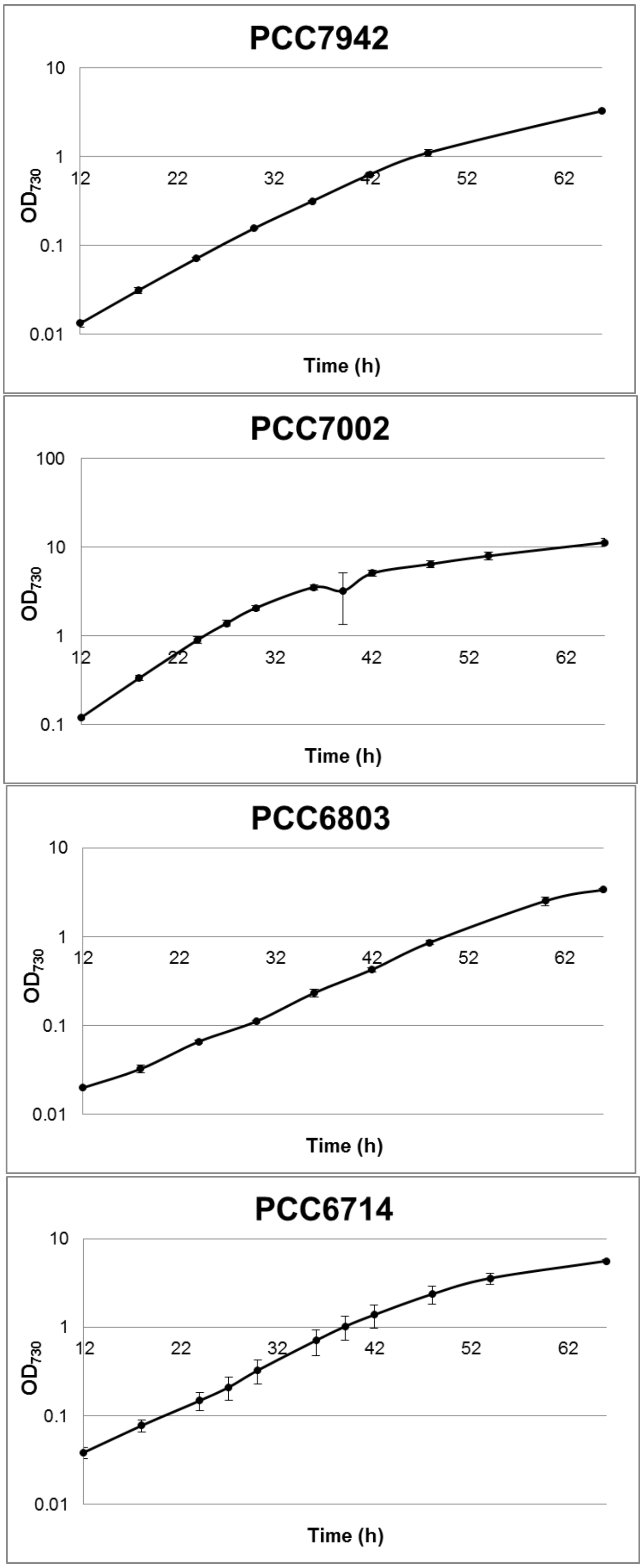

Table 2. The ratio of U-^{13}C to (U-^{13}C+U-^{12}C) peak area in internal standard.

Metabolite	U-^{13}C / (U-^{13}C+U-^{12}C)	Metabolite	U-^{13}C / (U-^{13}C+U-^{12}C)
2-Isopropylmalate	0.990	Glutamine	0.996
2-Oxoglutarate	0.973	Glycerate	0.815
3PGA	0.999	Glycine	0.906
4-Aminobutanoate	0.086	Glycolate	0.447
6-Phosphogluconate	0.971	GMP	1.000
Acetyl CoA	1.000	GTP	1.000
ADP	0.997	Guanosine	0.980
ADP-Glc	1.000	Histidine	0.398
Alanine	0.958	HMBPP	1.000
α-Glycerophosphate	0.997	Homoserine	0.952
AMP	1.000	Hydroxyproline	0.995
Arginine + Ornithine	0.998	IMP	1.000
Asparagine	0.992	Inosine	1.000
Aspartate	0.993	IPP + DMAPP	1.000
ATP	1.000	Isocitrate	0.905
Citrate	0.910	Isoleucine	0.943
CMP	1.000	Leucine	0.953
CTP	1.000	Lysine	0.623
Cystein	1.000	Malate	0.786
Cytidine	0.632	MEP	0.980
DHAP	0.996	Methionine	0.937
Disaccharide-P	1.000	Mn6P	1.000
F1P	0.991	NAD	1.000
F6P	1.000	NADP	1.000
FAD	1.000	Nicotinate	1.000
FBP	1.000	Orotate	0.971
FMN	1.000	Pantothenate	1.000
Fumarate	0.885	PEP	0.999
G1P	0.954	Phenylalanine	0.877
G6P	0.993	Proline	0.946
GAP	0.999	Pyridoxamine-5P	1.000
GDP	0.990	Pyroglutamate	0.951
Glutamate	0.999	Pyruvate	0.954
R5P	0.998	R1P	0.973
Ru5P	1.000	Tryptophan	0.799
RuBP	1.000	Tyrosine	0.990
S7P	1.000	UDP-Glc	1.000
Serine	0.881	UMP	0.922
Shikimate	1.000	Uridine	1.000
Succinate	0.881	UTP	1.000
Threonine	0.924	Valine	0.966
TMP	0.996	XMP	1.000

Figure 4. $\log_3$-$\log_3$ transformed liner calibration curve of each metabolite. Calibration curves were determined by least square linear regression analysis. The vertical axis represents naturally labeled standard amount in nmol per tube and the horizontal axis represents the area ratio of monoisotopic peak to uniformly ^{13}C-labeled peak.

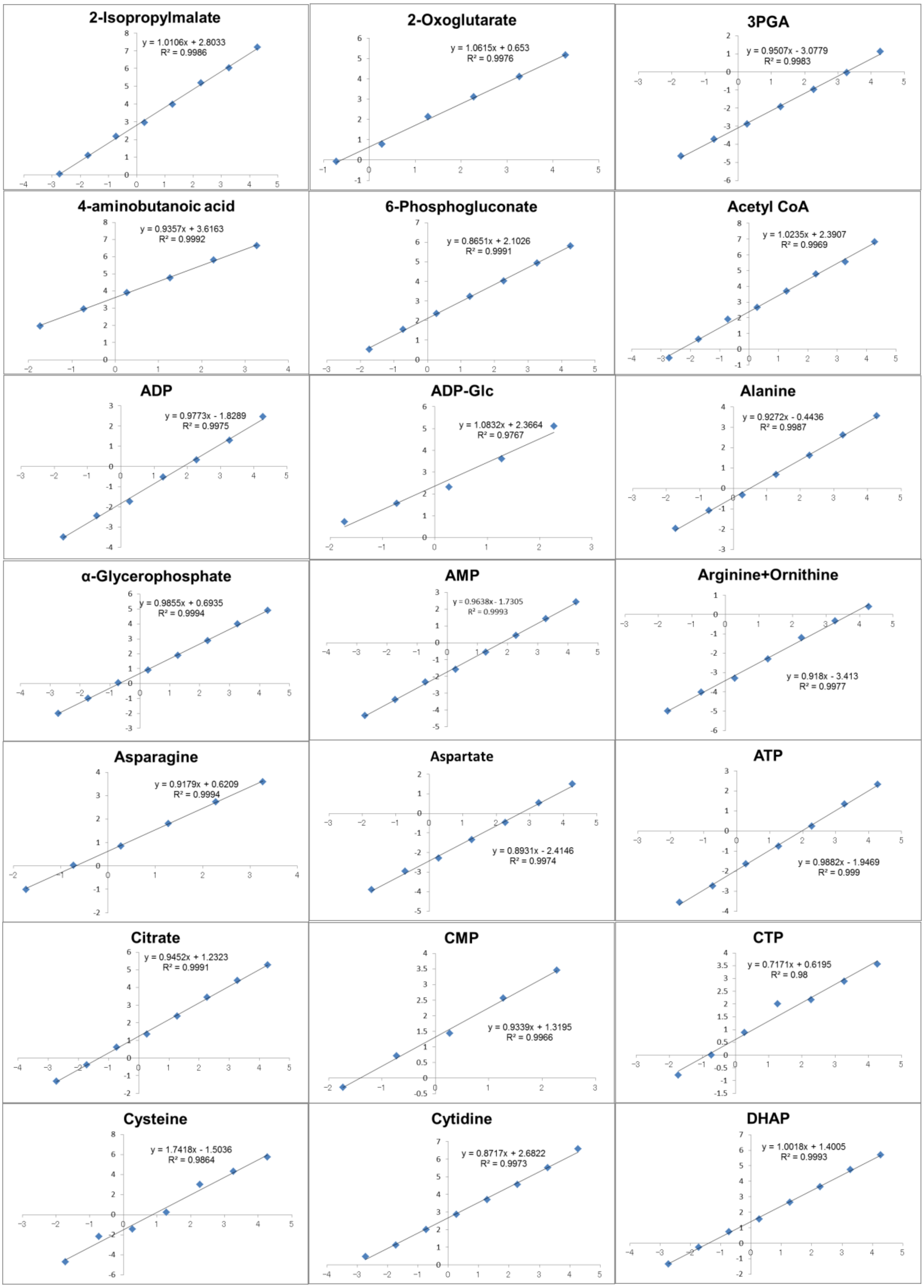

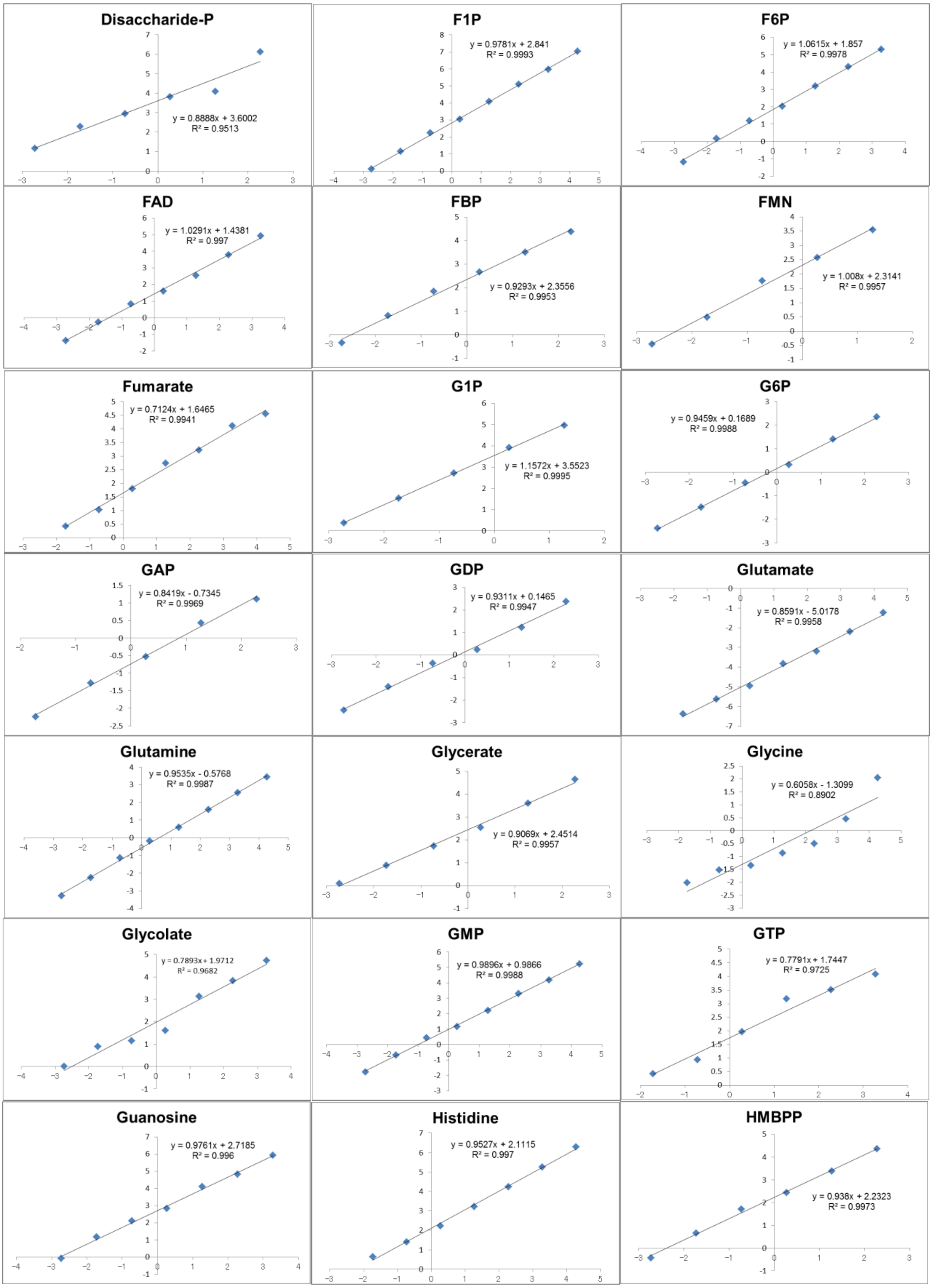
Disaccharide-P
y = 0.8888x + 3.6002
R² = 0.9513
F1P
y = 0.9781x + 2.841
R² = 0.9993
F6P
y = 1.0615x + 1.857
R² = 0.9978
FAD
y = 1.0291x + 1.4381
R² = 0.997
FBP
y = 0.9293x + 2.3556
R² = 0.9953
FMN
y = 1.008x + 2.3141
R² = 0.9957
Fumarate
y = 0.7124x + 1.6465
R² = 0.9941
G1P
y = 1.1572x + 3.5523
R² = 0.9995
G6P
y = 0.9459x + 0.1689
R² = 0.9988
GAP
y = 0.8419x - 0.7345
R² = 0.9969
GDP
y = 0.9311x + 0.1465
R² = 0.9947
Glutamate
y = 0.8591x - 5.0178
R² = 0.9958
Glutamine
y = 0.9535x - 0.5768
R² = 0.9987
Glycerate
y = 0.9069x + 2.4514
R² = 0.9957
Glycine
y = 0.6058x - 1.3099
R² = 0.8902
Glycolate
y = 0.7893x + 1.9712
R² = 0.9682
GMP
y = 0.9896x + 0.9866
R² = 0.9988
GTP
y = 0.7791x + 1.7447
R² = 0.9725
Guanosine
y = 0.9761x + 2.7185
R² = 0.996
Histidine
y = 0.9527x + 2.1115
R² = 0.997
HMBPP
y = 0.938x + 2.2323
R² = 0.9973

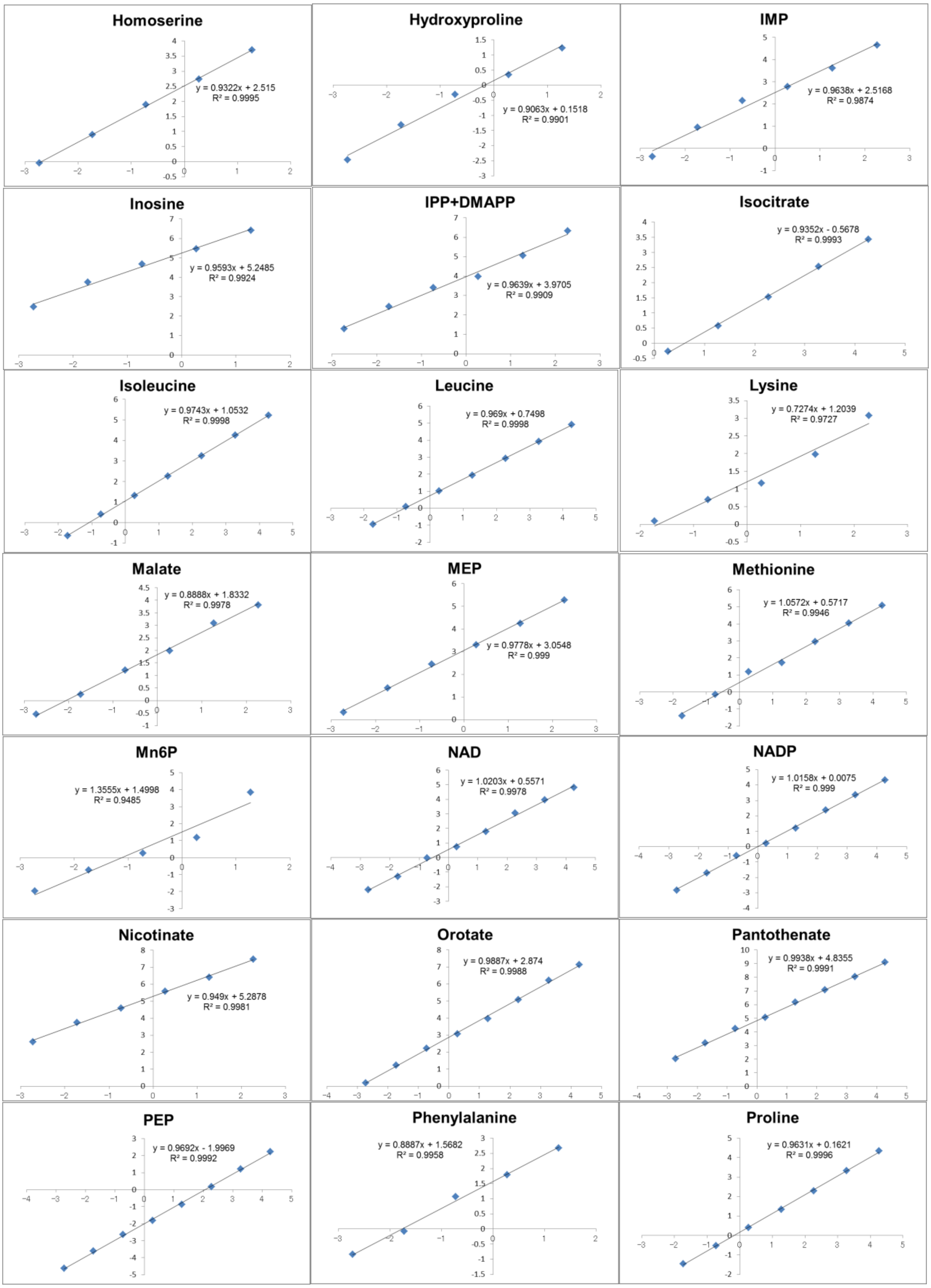
Homoserine
y = 0.9322x + 2.515
R² = 0.9995
Hydroxyproline
y = 0.9063x + 0.1518
R² = 0.9901
IMP
y = 0.9638x + 2.5168
R² = 0.9874
Inosine
y = 0.9593x + 5.2485
R² = 0.9924
IPP+DMAPP
y = 0.9639x + 3.9705
R² = 0.9909
Isocitrate
y = 0.9352x - 0.5678
R² = 0.9993
Isoleucine
y = 0.9743x + 1.0532
R² = 0.9998
Leucine
y = 0.969x + 0.7498
R² = 0.9998
Lysine
y = 0.7274x + 1.2039
R² = 0.9727
Malate
y = 0.8888x + 1.8332
R² = 0.9978
MEP
y = 0.9778x + 3.0548
R² = 0.999
Methionine
y = 1.0572x + 0.5717
R² = 0.9946
Mn6P
y = 1.3555x + 1.4998
R² = 0.9485
NAD
y = 1.0203x + 0.5571
R² = 0.9978
NADP
y = 1.0158x + 0.0075
R² = 0.999
Nicotinate
y = 0.949x + 5.2878
R² = 0.9981
Orotate
y = 0.9887x + 2.874
R² = 0.9988
Pantothenate
y = 0.9938x + 4.8355
R² = 0.9991
PEP
y = 0.9692x - 1.9969
R² = 0.9992
Phenylalanine
y = 0.8887x + 1.5682
R² = 0.9958
Proline
y = 0.9631x + 0.1621
R² = 0.9996

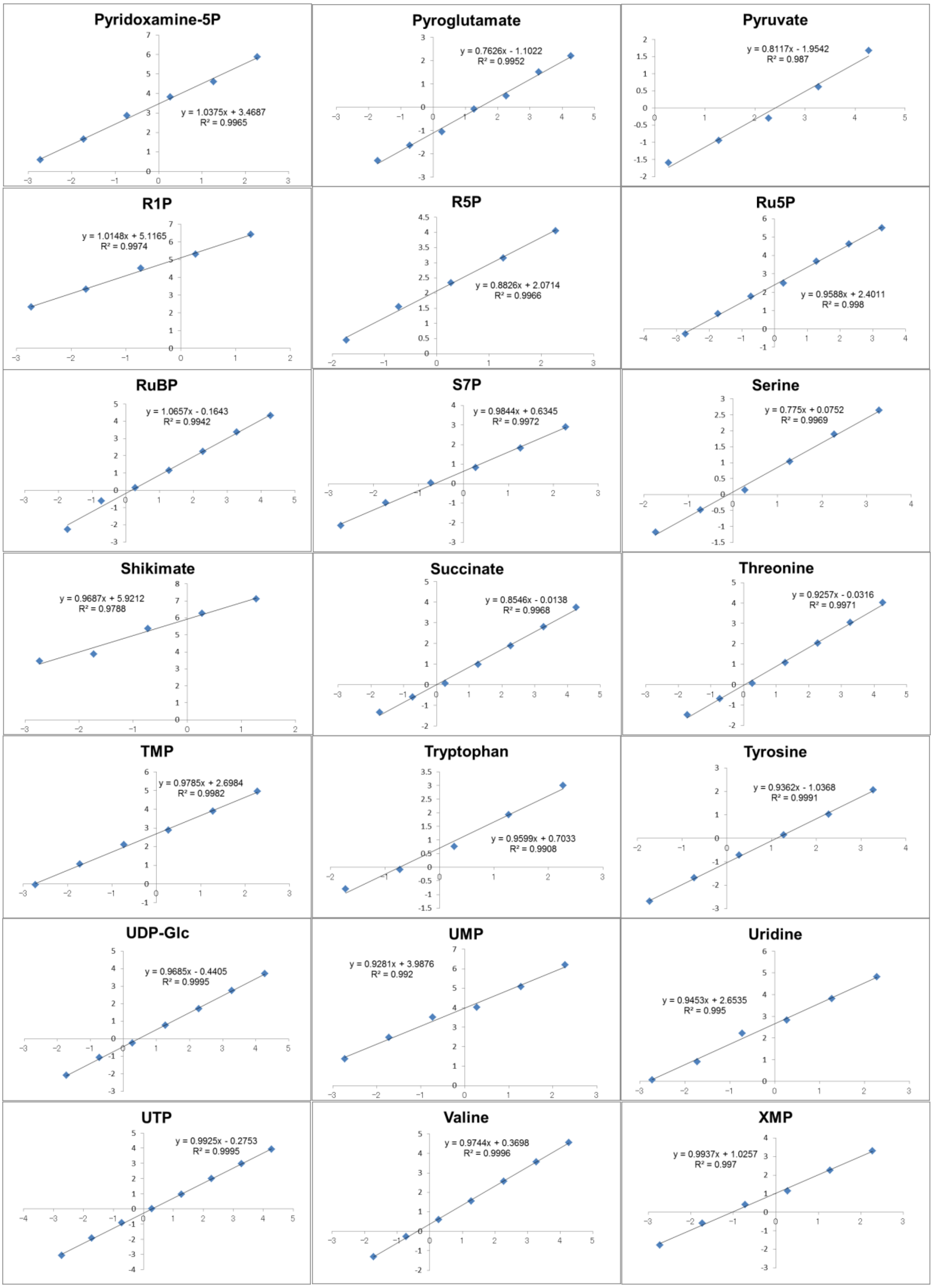
Pyridoxamine-5P
y = 1.0375x + 3.4687
R² = 0.9965
Pyroglutamate
y = 0.7626x - 1.1022
R² = 0.9952
Pyruvate
y = 0.8117x - 1.9542
R² = 0.987
R1P
y = 1.0148x + 5.1165
R² = 0.9974
R5P
y = 0.8826x + 2.0714
R² = 0.9966
Ru5P
y = 0.9588x + 2.4011
R² = 0.998
RuBP
y = 1.0657x - 0.1643
R² = 0.9942
S7P
y = 0.9844x + 0.6345
R² = 0.9972
Serine
y = 0.775x + 0.0752
R² = 0.9969
Shikimate
y = 0.9687x + 5.9212
R² = 0.9788
Succinate
y = 0.8546x - 0.0138
R² = 0.9968
Threonine
y = 0.9257x - 0.0316
R² = 0.9971
TMP
y = 0.9785x + 2.6984
R² = 0.9982
Tryptophan
y = 0.9599x + 0.7033
R² = 0.9908
Tyrosine
y = 0.9362x - 1.0368
R² = 0.9991
UDP-Glc
y = 0.9685x - 0.4405
R² = 0.9995
UMP
y = 0.9281x + 3.9876
R² = 0.992
Uridine
y = 0.9453x + 2.6535
R² = 0.995
UTP
y = 0.9925x - 0.2753
R² = 0.9995
Valine
y = 0.9744x + 0.3698
R² = 0.9996
XMP
y = 0.9937x + 1.0257
R² = 0.997
